# Supplementary material for: Investigating the relationship between intergroup physical contact and attitudes towards foreigners: the mediating role of quality of intergroup contact
Source: PeerJ. 2018 Sep 27;6:e5680. doi: 10.7717/peerj.5680 (PMC6164546; doi:10.7717/peerj.5680)
Supplement: Supplemental Information 1 [file peerj-06-5680-s001.docx]

We calculated the mean of each variable by aggregating all participants’ actual ratings (e.g., if a participants rated 2 out of 3 items of the quality of intergroup contact measure, the aggregate score for this participant would be calculated by averaging the ratings on 2 items only). We then performed the correlation analyses between the variables, resulting from the above-mentioned logic. Results largely overlapped the associations between variables presented in the main analyses of the previously submitted manuscript, and showed in Figure 1 both in terms of significance and coefficient size. Specifically, the association between EIPC and quality (*r* = .46, *p* <.001), EIPC and quantity (*r* = .63, *p* <.001), quality and prejudice (*r* = .57, *p* <.001), quantity and prejudice (*r* = .25, *p* <.001), and EIPC and prejudice (*r* = .27, *p* <.004) were all significant and in the expected direction*.*
